# Potential Benefits of Nitrate Supplementation on Antioxidant Defense System and Blood Pressure Responses after Exercise Performance

**DOI:** 10.1155/2019/7218936

**Published:** 2019-03-27

**Authors:** Eduardo F. Menezes, Leonardo G. Peixoto, Renata R. Teixeira, Allisson B. Justino, Guilherme M. Puga, Foued S. Espindola

**Affiliations:** ^1^Institute of Biotechnology, Federal University of Uberlandia, Uberlandia, MG, Brazil; ^2^Laboratory of Cardiorespiratory and Metabolic Physiology, Physical Education Department, Federal University of Uberlandia, Uberlandia, MG, Brazil

## Abstract

Nitrate (NO_3_^−^) supplementation is associated with exercise performance, oxygen uptake, blood flow, and blood pressure improvement, and it can act as an antioxidant agent. This study evaluated the effects of sodium nitrate supplementation on oxidative stress markers and blood pressure responses after aerobic exercise performance in physically active males. Fourteen subjects aged 22 ± 3 years and with a BMI of 23 ± 1 kg/m^2^ were submitted to four exercise tests in intervals of 5 days. Nitrate supplementation (NO session) and placebo supplementation (PL session) were acute (AC) and over a period of 5 days (FD) in random order with a crossover design. Saliva was collected at basal (0′); 60 min after supplementation (60′); immediately after exercise (90′); and 15, 30, and 60 min after the test (105′, 120′, and 150′). The NO session had higher concentrations (*P* < 0.05) of salivary nitrite in both AC and FD treatments when compared with the PL session. There was a reduction in systolic blood pressure (SBP) only after FD in the NO session. Furthermore, uric acid and total antioxidant capacity (FRAP) salivary concentrations increased, while SOD activity and TBARS levels decreased after FD but not after AC in the NO session. The results suggest that nitrate supplemented over a period of 5 days reduced SBP and indirectly acted as an antioxidant in healthy nonsedentary young men.

## 1. Introduction

The effect of nitrate supplementation on human health has been evaluated. Nitrate can be used as a substrate for the synthesis of nitric oxide (NO) from the nitrate-nitrite-NO pathway [1, 2]. Recently, our group and others have shown that nitrite can be used as an index of training intensity and load [3] in the reduction of blood pressure [4, 5], in the resting metabolic rate [6], and in the modulation of mitochondrial function [7]. In addition, nitrate supplementation decreases the oxygen cost of submaximal exercise and increases high-intensity exercise tolerance [8]. The increase in NO bioavailability acts on the relaxation of the vascular wall [9], as well as on the balance between antioxidants and prooxidant agents, improving vascular function [9]. These findings suggest improvement in vascular health, in addition to enhancing physical performance from increased NO bioavailability.

Besides NO formation, nitrate supplementation can act as an antioxidant potential agent, which suppresses free radical formation [10]. Oxidative stress is characterized as an imbalance between the increase in reactive oxygen species (ROS) and the ability of biological systems to decrease reactive intermediates [11]. Regarding its antioxidant role, NO can eliminate the oxidants produced by the Fenton reaction, reduce equivalents provided by superoxide (O_2_^–^), or prevent the reaction of peroxide [12]. Thus, sodium nitrate and other nitrate donors, such as beetroots, have been shown to suppress radical formation and to be scavengers of potentially damaging reactive oxygen and nitrogen species, suggesting nitrate may also exhibit antioxidant effects [10, 12].

Regarding nitrate supplementation and physical exercise, evidence indicates that an improvement in cardiovascular health and physical performance is plausible but inconsistent [13]. For example, the duration of the supplementation period and maintenance of a normal diet during the supplementation period can enhance exercise tolerance and performance [8, 14, 15] or not improve performance [16, 17], but this is by no means a universal finding. Likewise, there is little evidence on the influence of nitrate supplementation on oxidative stress in physical exercise. Carriker et al. [18] showed that acute dietary nitrate supplementation does not attenuate oxidative stress during submaximal exercise. However, no study showed the effect of chronic nitrate supplementation on oxidative stress after exercise. Therefore, considering that nitrite can increase dilatation of blood vessels and decrease free radical formation, in this study, we investigated the effect of sodium nitrate supplementation on oxidative stress markers and blood pressure responses after moderate-intensity aerobic exercise performance in physically active males.

## 2. Material and Methods

### 2.1. Volunteers

Fourteen healthy young men (22 ± 3 years of age, 69.3 ± 3 kg body mass, 23 ± 1 kg/m^2^ BMI, 56.9 ± 1.5 kg lean mass, 14.8 ± 1.5 kg fat mass, and 243 ± 11 W maximum workload) that were nonsmokers and not using any kind of supplementation were recruited. The volunteers were physically active but not highly trained in sports. An incremental test was performed to evaluate the maximal workload and to prescribe the main test intensity. The experimental protocol was approved by the local Institutional Review Board and is in accordance with the Declaration of Helsinki.

### 2.2. Design

**Figure 1:**
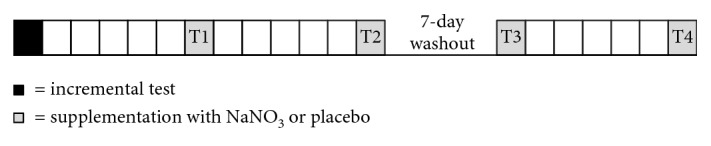
Experimental design. Each square represents 1 day. In the 1st day, an incremental test in the cycle ergometer was performed (black square). After 5 days, the acute test was performed (T1). Then, 5 days of supplementation with nitrate or placebo was carried out and the exercise was repeated (T2). After 7 days of washout, the experimental with crossover supplementation was repeated (T3, acute, and T4, 5 days of supplementation). Saliva was collected at basal (0′), 60 minutes after supplementation (60′), immediately after exercise (90′), and 15, 30, and 60 minutes after the test (105′, 120′, and 150′).

This study was conducted to investigate the acute and chronic effects of a 5-day supplementation of sodium nitrate or placebo in a crossover design and in random order for each volunteer. All volunteers were instructed to maintain their normal exercise and diet routines throughout the experimental period but to avoid alcohol, caffeine, and foods rich in nitrate 24 h prior to the tests.  Figure 1 shows the study design.

During the first visit, body composition was measured by bioelectrical impedance (BIA balance-pole, Tanita BC558) and the volunteers were familiarized with the cycle ergometer (Cycle Ergometer: Bike Mechanics Braking CEFISE, Campinas, SP). A maximal incremental test [19] was performed to identify the exercise intensity. The test consisted of applying 35-watt increments every 2 min and a fixed rotation of 70 rpm. The maximal workload was estimated according to the equation described by Stegmann and colleagues [20].

During the following 4 visits, both acute (AC) response (T1 and T3) and the response after 5 days with nitrate supplementation (FD) (T2 and T4) in moderate exercise performance were investigated. This intensity was chosen to allow the participant to perform at least 30 min on the bike, and it is well established to be moderate intensity that could promote both postexercise hypotension and enough oxidative stress [19]. The volunteers performed 30 min of cycle ergometer exercise with 50% maximum workload from 8:00–10:00 am. Nitrate supplementation (NaNO_3_ 10 mg kg^−1^ body weight, or placebo, sodium chloride in identical capsule) was carried out in both acute supplementation (AC: T1 and T3) and after 5-day supplementation (FD: T2 and T4). The washout period between T2 and T3 tests was 7 days in random order with a crossover design.

During the exercise tests, saliva samples were collected and heart rate (HR, Polar RS800CX) and blood pressure (BP, OMRON HEM 7200) were monitored at rest (0′); 1 h after supplementation (60′); immediately after the exercise test (90′); and 15 (105′), 30 (120′), 45 (135′), and 60 (150′) min after the test.

### 2.3. Saliva Collection

Saliva was collected in collection vials with no exogenous stimulation using the guidelines proposed by Granger et al. [21]. The subjects were instructed to avoid the ingestion of foods with high nitrite levels, such as green vegetables, beets, strawberries, grape, and tea, and the use of mouthwash during the entire study. They were also instructed to accumulate saliva, and the spit was deposited for 1 min and consequently centrifuged. The supernatant was stored at –80°C until the assay.

### 2.4. Nitrite (NO_2_^–^)

Nitric oxide bioavailability was estimated by the formation of nitrite (NO_2_^−^) in saliva using the Griess reaction [22]. Equal volumes of saliva and Griess reagent (1% sulfanilamide and 0.1% N-(1-naphthyl)ethylenediamine dihydrochloride in 2.5% phosphoric acid) were incubated. Nitrite content was calculated based on a standard curve of sodium nitrite (NaNO_2_). The concentration of total protein in each sample was determined using the Bradford method [23]. The analyses were performed using a VersaMax microplate reader (Molecular Devices, Menlo Park, CA, USA).

### 2.5. Alpha-Amylase

During salivary alpha-amylase (sAA) analysis, 10 *μ*g of total protein from each sample was loaded onto 5–22% SDS-PAGE [24]. Proteins were transferred onto nitrocellulose membranes (0.45 *μ*m) for 2 h at 100 mA at 4°C. Membranes were blocked for 4 h at 4°C in blocking buffer and incubated overnight at 4°C with a homemade affinity purified polyclonal rabbit anti-human sAA antibody, as reported in Santos et al. (2011). Membranes were subsequently incubated with secondary antibodies for 3 h, and alpha-amylase was detected using ECL reagents. Densitometry analyses of the spots were performed using ImageJ (US NIH, Bethesda, Maryland, USA).

### 2.6. Salivary Lactate

Salivary lactate was analysed by the electroenzymatic method using a biochemical analyser YSI 2300 Stat Plus (Yellow Springs, Ohio, USA).

### 2.7. Salivary Uric Acid

Salivary uric acid concentrations were measured with a kit supplied by Labtest (Labtest, Brazil). Uricase was used in the assay to transform uric acid into allantoin and hydrogen peroxide. In the presence of peroxidase, hydrogen peroxide reacts with 4-aminoantipyrine and DHBS, forming the chromogen antipirilquinonimine.

### 2.8. Total Antioxidant Capacity (FRAP)

Total antioxidant capacity was evaluated by assessing the reduction of iron in its ferric state (Fe^3+^) to its ferrous state (Fe^2+^) at low pH. Total antioxidant capacity was calculated based on a standard curve constructed using 6-hydroxy-2,5,7,8-tetramethylchroman-2-carboxylic acid (Trolox).

### 2.9. Thiobarbituric Acid (TBARS)

Lipid peroxidation was assayed by measuring TBARS products using the method described by Walls et al. (1976) and adapted for a microplate format. Trichloroacetic acid (50% *w*/*v*) was added to the saliva supernatant, followed by incubation and centrifugation. Subsequently, the supernatant was removed and 0.75% thiobarbituric acid and 0.75% in 0.1 M HC1 were added. The samples were heated at 90–95°C for 20 min and centrifuged. The standard curve was prepared with malonaldehyde bis(dimethyl acetal) hydrolysed with 6 M HC1.

### 2.10. Superoxide Dismutase (SOD)

Superoxide dismutase (SOD) activity was kinetically evaluated according to the inhibition of the reaction of superoxide radical with pyrogallol. The SOD activity was determined by measuring the oxidized pyrogallol formation rate.

### 2.11. Statistical Analyses

Data was tested for normality using the Shapiro–Wilk test prior to the analyses. All values at each sampling time were averaged and compared between sessions using one-way ANOVA, followed by Tukey's posttest for parametric values and the Kruskal–Wallis test, followed by Dunn's test for nonparametric values. The area under the curve (AUC) was used as temporal analysis of the variables. For all analyses, the significance level was set at *P* < 0.05. Results are shown as means ± standard error mean (SEM).

## 3. Results

**Figure 2:**
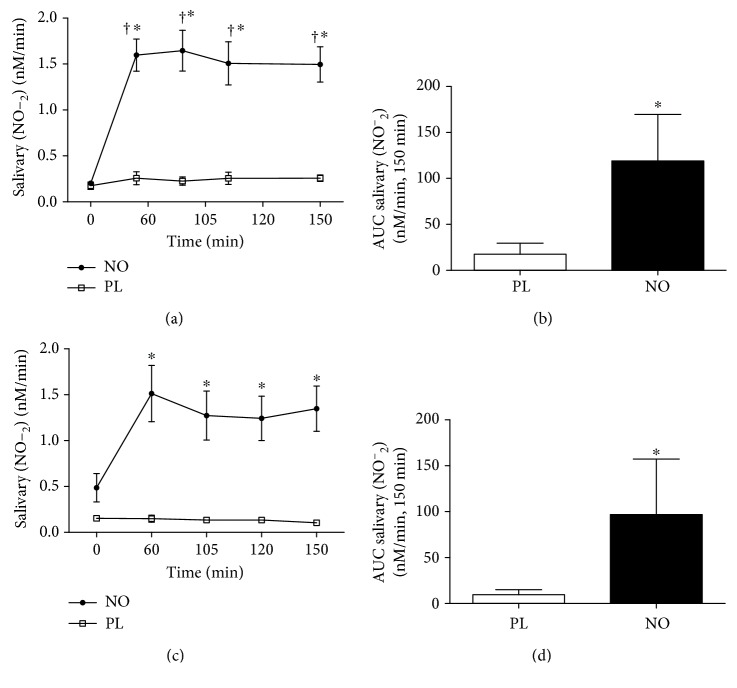
Concentration of salivary nitrite post 30 min of exercise on cycle ergometer. (a) Acute supplementation. (b) AUC postexercise with acute supplementation. (c) Five days of supplementation. (d) AUC postexercise with five days of supplementation. ^†^Difference from the baseline session (*P* < 0.05); ^∗^statistical difference from the PL session (*P* < 0.05).

The habitual physical activity program and dietary intake among all subjects was similar during the experimental period. As expected, no side effects were reported, and salivary nitrite concentration increased after 60 min of nitrate supplementation. This increase remained 150 min postsupplementation (Figures 2(a) and 2(c)). In addition, 5 days of nitrate supplementation increased nitrite salivary in NO, as compared to the PL group in preexercise. No difference in nitrite concentration was observed postexercise (60′–150′) compared to preexercise (0′) in the NO group (Figure 2(c)). AUC in postexercise was higher in NO than in PL in both the acute and 5-day supplementations (Figures 2(b) and 2(d)).

**Figure 3:**
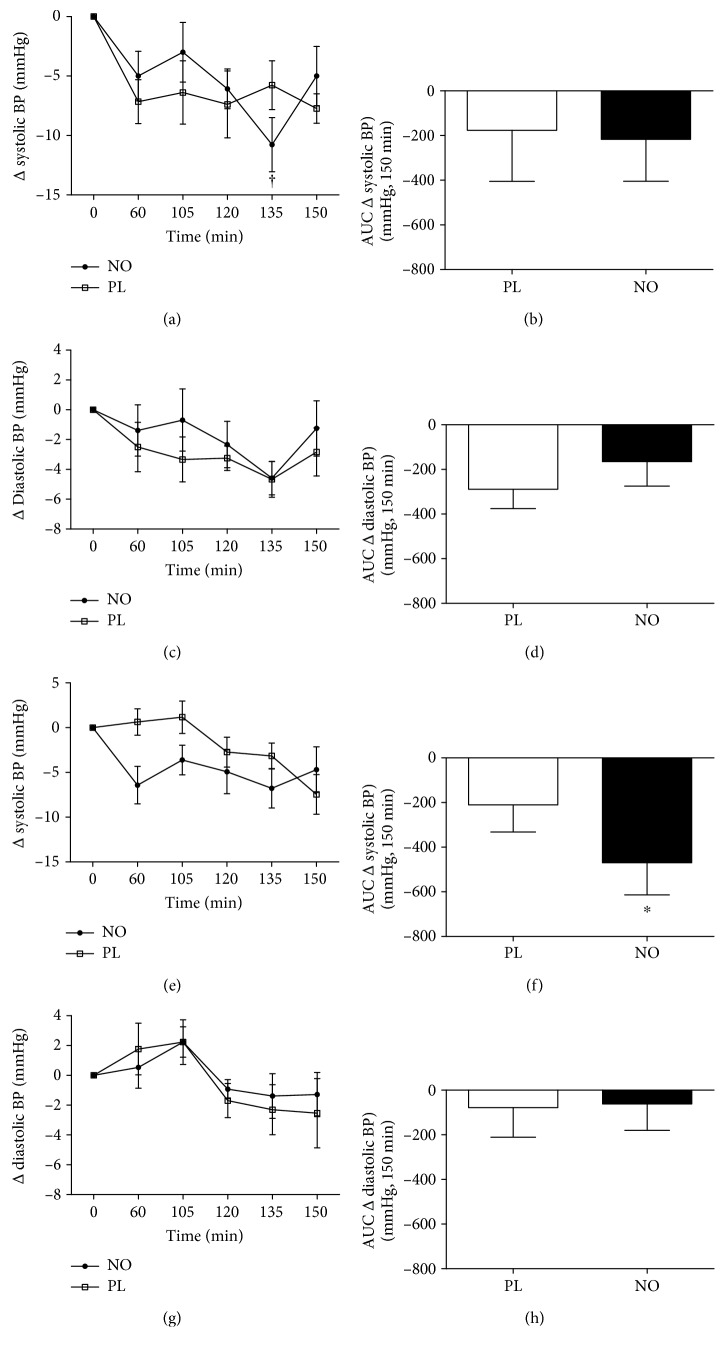
Systolic and diastolic blood pressure changes after 30 min of cycle ergometer exercise in acute and after 5 days of supplementations. Acute supplementation (a–d). Five days of supplementation (e–h). AUC after exercise (b, d, f, and h). ^∗^Statistical difference from the PL session (*P* < 0.05).

Systolic and diastolic blood pressure profiles are shown in Figure 3. The AUC of systolic blood pressure in postexercise decreased (*P* < 0.05) only after 5 days of nitrate supplementation in NO compared to the PL group (Figure 3(f)) but did not change after acute supplementation (AC). No differences were observed in diastolic blood pressure after acute or 5-day supplementations (Figures 3(g)–3(h)). It was also found that salivary NO^2−^ was higher (*P* < 0.05) in the baseline at time 0 after FD (data not shown in Figure 3(c)).

**Figure 4:**
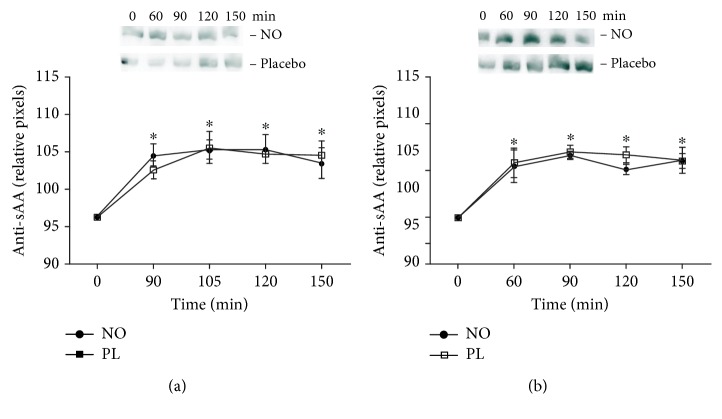
Salivary alpha-amylase (sAA) content after 30 min of cycle ergometer exercise. (a) Acute supplementation. (b) Five days. ^∗^Statistical difference from preexercise (0′) (*P* < 0.05).

Salivary alpha-amylase content increased after exercise in both acute and 5-day supplementations as compared to baseline (0′). No difference was observed in the sAA content after 30 min of cycle ergometer exercise in both the NO and the placebo groups (Figures 4(a) and 4(b)). In addition, salivary lactate increased after 30 min (90′ times) of cycle ergometer exercise, returning to baseline after 15′ of rest (Table 1).

**Figure 5:**
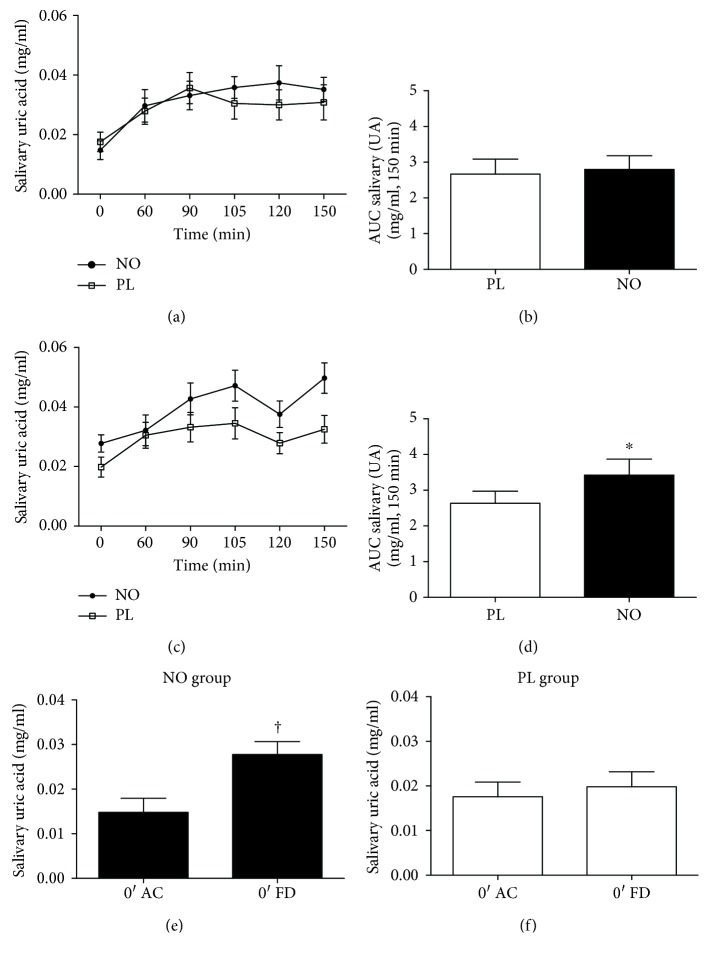
Salivary uric acid concentration after 30 min of cycle ergometer exercise in NO and PL group. (a) Acute supplementation. (b) AUC of acute supplementation. (c) Five days of nitrate supplementation. (d) AUC 5 days of nitrate supplementation. (e) Baseline uric acid concentrations from NO group. (f) Baseline uric acid concentrations from the PL group. ^∗^Statistical difference from the PL group (*P* < 0.05). ^†^Statistical difference in basal uric acid concentration between FD and AC treatments (*P* < 0.05).

Regarding the antioxidant potential of nitrate supplementation, the AUC of salivary uric acid concentration increased after 5 days of supplementation in NO compared to the PL group (Figure 5(d)). In addition, 5 days of nitrate supplementation increased the AUC of salivary uric acid in NO compared to the PL group preexercise (Figure 5(e)).

**Figure 6:**
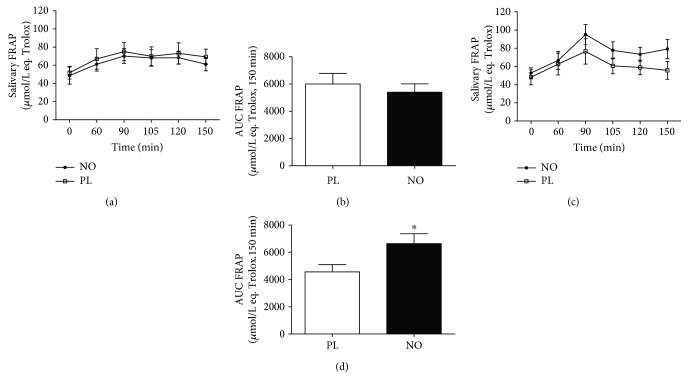
Total antioxidant capacity (FRAP) after 30 min of cycle ergometer exercise. (a) Acute supplementation. (b) AUC of acute supplementation. (c) Five days of supplementation. (d) AUC of 5 days of supplementation. ^∗^Statistical difference from the PL session (*P* < 0.05).

**Figure 7:**
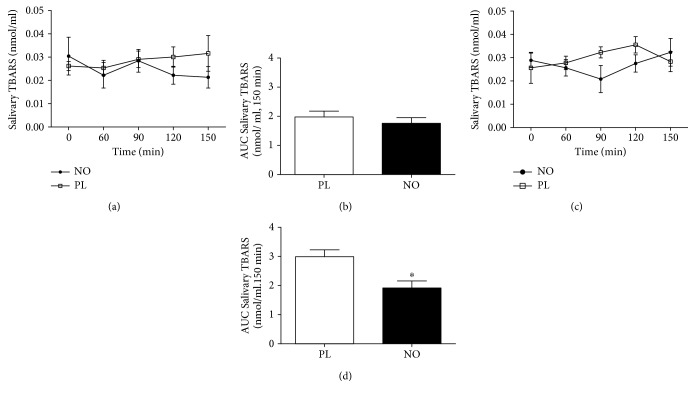
Salivary lipid peroxidation (TBARS) after 30 min of cycle ergometer exercise. (a) Acute supplementation. (b) AUC of acute supplementation. (c) Five days of supplementation. (d) AUC of 5 days of supplementation. ^∗^Statistical difference from the PL session (*P* < 0.05).

**Figure 8:**
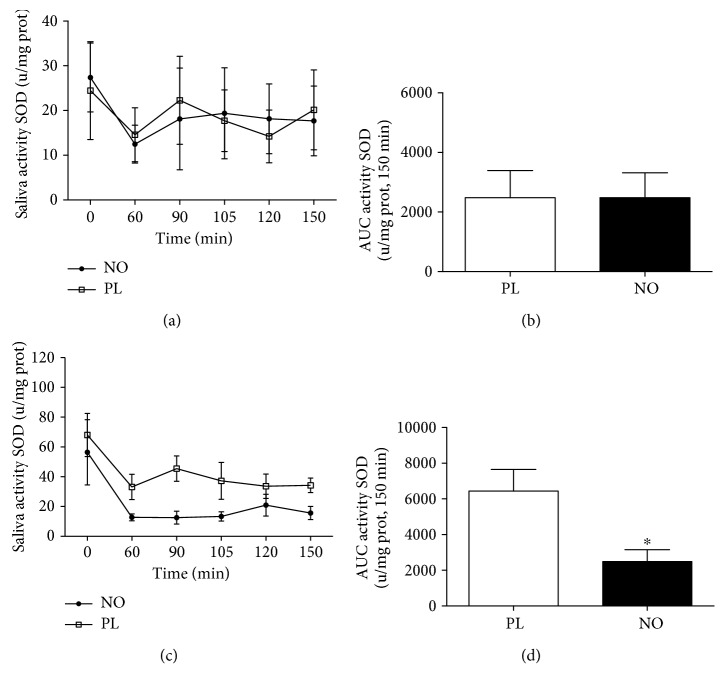
Salivary superoxide dismutase (SOD) activity after 30 min of cycle ergometer exercise. (a) Acute supplementation. (b) AUC of acute supplementation. (c) Five days of supplementation. (d) AUC of 5 days of supplementation. ^∗^Statistical difference from the PL session (*P* < 0.05).

When the total antioxidant capacity was analysed, 5 days of nitrate supplementation increased the AUC in NO compared to the PL group after 30 min of exercise (Figure 6(d)). In addition, 5 days of nitrate supplementation decreased the AUC in NO compared to the PL group after exercise (Figure 7(d)). In the same way, 5 days of nitrate supplementation decreased the AUC of SOD activity in NO compared to the PL group (Figure 8(d)).

## 4. Discussion

The present study investigated the effect of nitrate supplementation on blood pressure responses and antioxidant status after 30 min of aerobic exercise on cycle ergometer performance in trained subjects. The major results showed that 5 days of nitrate supplementation decreased systolic blood pressure and improved antioxidant response after aerobic exercise performance, as compared to acute or placebo supplementation.

As expected, nitrate supplementation increased the salivary nitrite concentration in both acute and 5-day supplementations. The daily nitrate dose used in this study corresponds to the amount normally found in 150 to 250 g of nitrate-rich vegetables, such as spinach, beetroot, or lettuce [25].

The intensity of exercise can be evaluated from an increase in salivary lactate [26], salivary alpha-amylase concentration, and salivary nitrite [3, 27]. Here, 50% maximum workload during 30 min of cycle ergometer exercise could increase both the salivary lactate and sAA content. However, compared to other experimental designs, the increase in salivary lactate and sAA found in our results was lower than that in exercises with 75% maximum workload [3]. Therefore, we consider that the 30 min of cycle ergometer exercise was of moderate intensity. In addition, our experimental group was composed of trained subjects, and after the first visit in our lab, these volunteers continued with the training load until returning to the lab.

Nitrate supplementation may reduce blood pressure after exercise performance [4, 5, 28]. These findings corroborate with the results of this study, which showed a decrease in systolic blood pressure AUC after exercise performance. The increase in salivary nitrite is related to the nitrate-nitrite-NO pathway [29], which can improve vascular health [30]. This blood pressure-lowering effect can be attributed to NO synthesis [31], which plays an important role in vascular health. This finding indicates that 5 days of nitrate supplementation can improve blood pressure responses mediated by aerobic exercise performance. This is important data because most studies have shown only blood pressure responses after nitrate supplementation alone, or after exercise training, but not after 90 min of exercise performance, which is well-described in the literature as an important tool to control blood pressure and prevent peaks of blood pressure and cardiovascular events [5, 15].

Regarding the antioxidant role of nitrate supplementation, the results showed an increase in uric acid in the NO group after 5 days of nitrate supplementation. Uric acid is the most important antioxidant molecule in saliva [32, 33]. In the extracellular environment, urate can scavenge hydroxyl radicals, singlet oxygen, and peroxynitrite, especially when combined with ascorbic acid or thiols [34]. Similar to the increased salivary uric acid, these results showed an increase in total antioxidant capacity (FRAP) only after 5 days of nitrate supplementation. Approximately 60% of total antioxidant capacity measured with the FRAP test was from uric acid [35], indicating uric acid is an antioxidant agent. In addition, FRAP reflects the ability to keep a balance between oxidants and antioxidants or even the ability to repair damage caused when the production of oxidants is found in greater proportions [36]. Here, lipid peroxidation (TBARS) and SOD activity decreased after 30 min of cycle ergometer exercise only in the group that received 5 days of nitrate supplementation. One possible explanation to improve lipid peroxidation is the increase in NO [37] from the nitrate-nitrite-NO pathway [1, 2]. Nitric oxide acts as a potent inhibitor of the lipid peroxidation chain reaction by scavenging propagatory lipid peroxyl radicals [12, 38, 39]. In addition, in the acute group, the nitrate supplementation did not attenuate oxidative stress. These results corroborate an earlier study, which showed that acute dietary nitrate supplementation did not attenuate oxidative stress [18]. Therefore, chronic nitrate supplementation can be an alternative method for improving oxidative stress after physical exercise.

As limitations of this study, we considered the absence of measures of vascular function and only measurements of antioxidant biomarkers in saliva. Also, we showed that total antioxidant capacity data is in consonance with uric acid levels, the main antioxidant in saliva, although only the FRAP method was used to evaluate the total antioxidant capacity. In addition, biomarker analysis in salivary fluid has practical appeal, as reported in many studies revealing systemic biomarkers of health status, and those specifically related to oxidative stress and antioxidant status are still needed.

## 5. Conclusion

Taken together, this study showed that 5 days of sodium nitrate supplementation reduced systolic blood pressure and improved antioxidant responses after moderate-intensity aerobic exercise in healthy and physically active men.

## Figures and Tables

**Table 1:** Salivary lactate concentration (*μ*m/L) in rest and post 30 min of cycle ergometer exercise.

NO group	0′	60′	90′	105′	120′	150′
	0.34 ± 0.18	0.39 ± 0.11	0.69 ± 0.29^∗^	0.53 ± 0.24	0.38 ± 0.18	0.43 ± 0.18

PL group	0′	60′	90′	105′	120′	150′
	0.34 ± 0.23	0.37 ± 0.24	0.66 ± 0.31^∗^	0.32 ± 0.24	0.40 ± 0.23	0.32 ± 0.20

^∗^Statistical difference from rest (*P* < 0.05).

## Data Availability

The results' data used to support the findings of this study are restricted by the local ethics board Comitê de Ética em Pesquisa em Seres Humanos from Federal University of Uberlandia in order to protect patient privacy. Data are available from Foued Salmen Espindola, Federal University of Uberlandia, Institute of Genetics and Biochemistry (INGEB), Rua Acre, S/N, Bloco 2E, Sala 237, Campus Umuruama, CEP 38400-902, Uberlandia, MG, Brazil (e-mail: foued@ufu.br, for researchers who meet the criteria for access to confidential data).
